# Systematic review and meta-analysis of lactose digestion, its impact on intolerance and nutritional effects of dairy food restriction in inflammatory bowel diseases

**DOI:** 10.1186/s12937-016-0183-8

**Published:** 2016-07-13

**Authors:** Andrew Szilagyi, Polymnia Galiatsatos, Xiaoqing Xue

**Affiliations:** 1 Division of Gastroenterology, Department of Medicine, Jewish General Hospital, Room E-110,3755 Cote Ste Catherine Rd, Montreal, QC H3T 1E2 Canada; 2 Department of Emergency Medicine, Jewish General Hospital, McGill University, Montreal, QC Canada

**Keywords:** Lactose malabsorption, Intolerance, Inflammatory Bowel Diseases

## Abstract

**Background:**

Relationships between inflammatory bowel disease and lactose containing foods remain controversial and poorly defined regarding symptoms, nutritional outcomes, and epidemiologic associations for lactose maldigestion.

**Methods:**

A literature review was performed using Pub Med, Cochrane library and individual references, to extract data on lactose maldigestion prevalence in inflammatory bowel diseases. A meta-analysis was done using selected articles, to determine odds ratios of maldigestion. Information was collected about symptoms, impact on pattern of dairy food consumption, as well as the effects of dairy foods on the course of inflammatory bowel diseases.

**Results:**

A total of 1022 articles were evaluated, 35 articles were retained and 5 studies were added from review articles. Of these 17 were included in meta-analysis which showed overall increased lactose maldigestion in both diseases. However increased risk on sub analysis was only found in Crohn’s in patients with small bowel involvement. Nine additional studies were reviewed for symptoms, with variable outcomes due to confounding between lactose intolerance and lactose maldigestion. Fourteen studies were evaluated for dairy food effects. There was a suggestion that dairy foods may protect against inflammatory bowel disease. Nutritional consequences of dairy restrictions might impact adversely on bone and colonic complications.

**Conclusions:**

Lactose maldigestion in inflammatory bowel disease is dependent on ethnic makeup of the population and usually not disease. No bias of increased disease prevalence was noted between lactase genotypes. Intolerance symptoms depend on several parameters besides lactose maldigestion. Dairy foods may decrease risks of inflammatory bowel disease. Dairy restrictions may adversely affect disease outcome.

## Introduction

The Inflammatory Bowel Diseases (IBD), Crohn’s Disease (CD) and Ulcerative Colitis (UC), are complex conditions with enigmatic causes. Pathogenesis implicates interactions between a genetically susceptible host and a disturbed bacterial microflora resulting in aberrant innate and adaptive immune responses [1]. The intestinal microflora is responsive to various factors such as antibiotics and diet [2, 3]. In IBD, diet may be important both for pathogenesis and nutrition [4–6], although specific proof is lacking for the former [7].

The role of dairy foods (DFs) in IBD has been controversial and confounded by the phenotypic divide of lactase status in the adult population. About 1/3 of adults retain the ability to digest lactose (LP; lactase persistence, lactose digesters) while the rest lose it (LNP; lactase non -persistence, lactose maldigesters [LM]). The links between lactose, milk, DFs and IBD are topics related on several levels. The world segregation into LP /LNP correlates with a number of diseases, including IBD, raising the question of a coincidental event or an evolutionary modifier of disease similar to latitudinal distributions [8]. As such, unequal phenotype distributions of LP/LNP in IBD may be an additional risk factors for IBD [9] or may predispose to LM. Patients with IBD may find that DFs aggravate their symptoms, leading them and some professionals to recommend a reduced lactose diet [10]. In healthy persons milk and other DF avoidance is partly related to true lactose intolerance (LI) or the presumption of LI due to suggestive symptoms [11, 12]. However symptoms from diet are also affected by consumption of Fermentable Oligo, Di, Monosaccharide And Polyols (FODMAPs) in IBD [13, 14]. Lactose is generally excluded in a low FODMAP diet independent of lactose digestion status.

It also remains unclear what role DF avoidance has on nutritional effects on patients with IBD. This systematic review seeks primarily to determine the prevalence of LM in IBD and establish whether there is a bias toward either phenotype. Secondary outcomes were determining whether symptoms of LI play a role in DF avoidance, and whether DF restriction impacts on IBD course.

## Methods

### Search strategy

A review of the literature between Jan 1965 to June 2016 was undertaken. The search engines Medline (Pub Med) and Cochrane Library were used to obtain relevant articles. Terms used were lactose maldigestion or lactose intolerance or milk intolerance or lactose sensitivity (LI with systemic symptoms) AND Inflammatory Bowel Disease or Crohn’s disease or Ulcerative colitis. In the case of the Cochrane library, the terms “systematic review” or “meta-analysis” were also selected, to narrow the search. Two authors (AS and PG) independently evaluated articles for inclusion in meta-analysis and disagreement was settled by consensus. A second search for articles was also included with the terms nutritional benefits OR detriments of milk OR dairy products in Inflammatory Bowel Disease, Chrohn’ disease Or Ulcerative colitis. References of individual review articles were also screened for relevant publications.

### Definitions

For the purpose of meta-analysis a clear distinction was made between objective tests of LM vs. symptoms attributed to LI or sensitivity. The reasons for this are that LI is subjective and can occur in the absence of LM and symptoms elicited during lactose challenge tests do not necessarily reflect reactions to DF ingestion. The term lactose tolerance test (LTT) retains the name but an abnormal test suggests LM and LI may occur as with the hydrogen breath test (BT). Small bowel biopsies, urinary sugar tests or genetic tests define the propensity for LM but do not predict symptomatic LI.

### Article eligibility for meta-analysis or nutritional effects

Original articles and case reports (including more than 5 patients) were included if patients underwent objective testing for lactose digestion (regardless of method), and if they were compared to a healthy control group. Abstracts in English were included if sufficient data were available from the report. Articles including patients with other diseases but no IBD were excluded from analyses. For the second outcome, looking at prevalence of symptoms of LI in IBD, articles referring to LI, or DF intolerance or sensitivity were also sought, regardless of formal testing for lactose digestion. For nutritional impact, studies investigating DF effects or general diet on IBD were sought. The latter had to include reference to milk or DFs. Additional references were manually extracted from review articles on the topic.

### Data extraction for meta-analysis

Year of publication, country of origin, number of patients and controls, type of test for LM, test outcomes, distribution of CD or UC cases, site of involvement in CD, disease activity at time of testing, and surgical history were recorded from each study. A description of genetic likelihood of LM for patients versus controls was estimated. Each study included in the meta-analysis was graded by country for low (= grade 1, ≤20 % LNP), moderate (= grade 2, 21 –79 % LNP), or high risk (=grade 3, LNP ≥80 %), based on classification as per Mishkin [15] and Szilagyi [9].

### Quality assessment

Articles included in the meta-analysis were graded based on the Newcastle Ottawa scale for case control studies [16]. In this scheme, high quality studies achieved a score of 5 or more, and scores of 4 or less were considered low quality. Abstracts were not graded. PRISMA guidelines were followed [17].

### Data analysis

For each study, two by two tables of LM status (LM vs. not-LM) and IBD status (IBD vs. healthy control) were obtained. For studies with a zero cell, a continuity correction of 0.5 was used [18]. The association between LM and IBD were assessed using odds ratios (OR) and the corresponding 95 % confidence intervals (CIs). An OR greater than 1.0 indicates an increased risk of LM among IBD group compared with the healthy control group. The statistical significance of the summary OR was determined with the Z test, and a *p*-value less than 0.05 was considered statistically significant.

The heterogeneity among studies was determined by the Cochran Q statistics, where a *p*-value greater than 0.05 indicates a lack of heterogeneity. The *I* ^2^ statistics were also presented [19]. For the qualitative interpretation of heterogeneity, *I* ^2^ values of at least 50 % are usually considered to represent substantial heterogeneity, while values of at least 75 % indicate considerable heterogeneity according to the Cochrane Handbook. The summary OR was obtained using a fixed-effect model (Mantel-Haenszel method) when there was a lack of heterogeneity (*I* ^2^ ≤ 50 %), or a random-effects model (the DerSimonian and Laird method) when otherwise [20, 21]. The potential publication bias was estimated by a funnel plot for the overall analysis. Egger’s linear regression test on the natural logarithm scale of the OR was used to assess the funnel plot asymmetry; the significance was set at the *p* < 0.05 [22].

Sensitivity analysis was performed according to the following subgroups: type of IBD (CD or UC), specific disease site in the case of CD (SB-small bowel only, TiC-Terminal ileum and Colon, DC-colon only); type of test (BT-Breath Hydrogen measurement, LTT-Lactose tolerance test, urinary sugar ratios and small bowel biopsies); and finally evaluation of LM among the low risk group. The purpose of analyzing low risk group separately was that any disease effect involving intestinal lactase levels would be more likely to be detected in this group. All analyses were performed using SAS statistical package, version 9.1 (SAS Institute Inc., Cary, NC, USA).

## Results

**Fig. 1:**
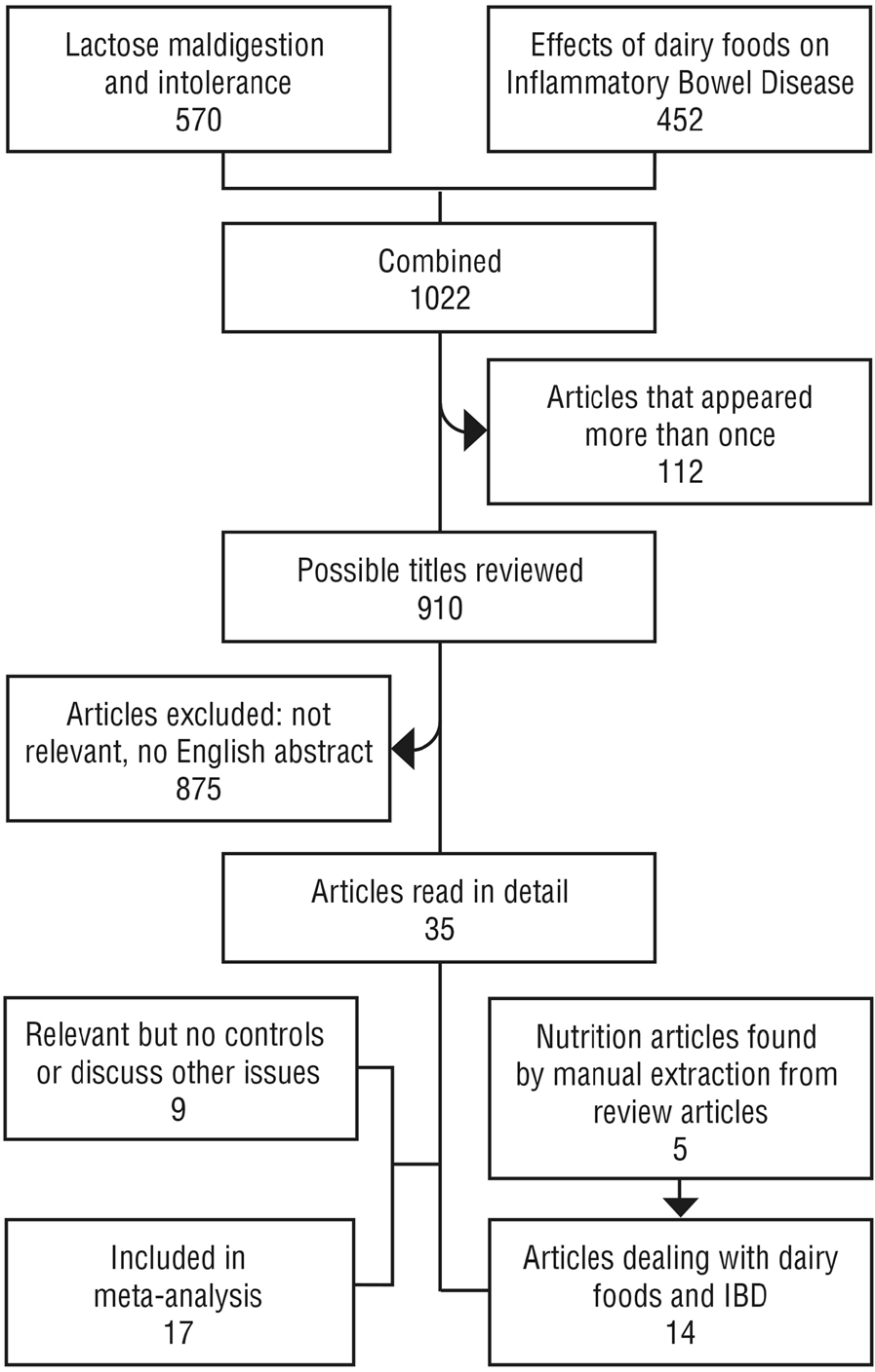
The outline for flow of retrieved articles

The first search yielded a total of 570, while the complementary search yielded an additional 452 publications during the specified time period. Of the combined 1022 articles, 35 studies were retained, as per inclusion/exclusion criteria. Seventeen of these were included in the meta-analysis of prevalence of LM. Nine of 35 additional papers without controls, discussion of methodology or DF related symptoms were included for narrative review. Nine of 35 studies were included in a review of nutritional effects of DFs. A further 5 were added to these 9 after manual extraction from general diet review articles in IBD for a total of 40 papers (Fig. 1).

### Prevalence of lactose maldigestion in IBD

#### Description of included studies for meta-analysis

The meta-analysis included a total of 1935 IBD patients (560 CD, 614 UC) and 761 controls. Table 1 outlines demographics of 17 studies (23 - 38). The mean age of participants based on available data was 35.6 years for CD, 40.8 years for UC, and 37.7 years for controls. One study focused exclusively on a pediatric population (mean age 13.5, range of 5–18) [30]. Based on reports that specified gender distribution, there were more females in the IBD groups than the control group.

**Table 1:** Studies of lactose maldigestion in IBD using breath test, lactose tolerance test, jejunal biopsies or urinary sugar ratio tests

Author (reference) County	Activity of disease	Test Method	Age (female %)	CD N/ LM	Age (female %)	UC N/ LM	Age (female %)	Control N / LM	NOS Score
Gupta [33] India	I	BT	34.7 (26)^a^	27 / 10^a^	36.2	18 / 10^a^	36.2 (26)^a^	45 / 12	7
Eadala [24] Wales UK	I^c^	BT Gen	19–86 (38)	70^d^ / 2^b^	20–81 (44)	95 / 11^b^	21–56 (15)	30 / 0	8
Barrett [25] Australia	I^c^	BT	40 (56)	92^d^ / 39	40 (44)	56 / 22	34 (20)	71 / 13	9
Banos Madrid [34] Spain	nd	BT	nd	18 / 3	nd	24 / 4	nd	25 / 5	7
Ginard [35] Spain	nd	BT Quest	nd	nd	40 (32)	52 / 13	41 (20)	34 / 11	7
Von Turpitz [26] Germany	24A 25I^c^	BT Bx	39.9 (30)	49 / 16	nd	nd	43.1 (11)	24 / 5	6
Mishkin [15] Canada^f^	I^c^	BT	36.9 (62)	121^d^ / 70	37 (71)	139 / 65	nd nd	158 / 46	8
Bernstein [27] Canada^f^	18A 11I	BT Quest	nd	nd	39 (13)	29 / 13	41 (4)	14 / 5	8
Kochlar [36] India	22A 38I	BT	nd	nd	36.7 (35)	60 / 25	36.4 (7)	20 / 8	7
Ogata [38] Japan	nd	BT LTT	nd	32/29BT32/30LTT	nd	nd	nd	51/26BT51/37LTT	nd
Park [28] Scotland	21A 41I	Bx	40 (38)	62^d^	nd	nd	40 (9)	13	5
Lobely [29] England	nd	USR	42 nd	16	42 (nd)	6	28.5 (13)	40	6
Pironi [37] Italy	I^c^	BT	33 (23)	37 / 18	nd	nd	35 (36)	67 / 11	7
Kirschner [30] United States^f^	37A^g^ 33I of IBD	BT	13.5 (nd)	50 / 17	13.5 (nd)	20 / 5	nd (nd)	42	7
Pena [23] England	37A 35I	Bx	nd	nd	nd	72 / 9	nd	21 / 2	6
Tandon [31] United States^f^	nd	LTT	nd	nd	nd	70^e^ [51/12] [19/16]	nd	94^e^ [53/11] [41/29]	7
Chalfin [32] United States	nd	LTT	40.8 (2)	5 / 3	42.8 (5)	9 / 4	44.3 (7)	12	5

*BT* breath hydrogen test, *LTT* lactose tolerance test, *Bx* jejunal biopsy, *USR* urinary sugar ratio(lactose/L-arabinose), *Gen* Genetic test for north European lactase polymorphism C/T-13910, *Quest* questionnaire, *LM* lactose maldigestion, *LA* lactose absorption, nd-not done or not stated, *I* inactive disease, *A* active disease

^a^Gupta reported LM % and gender distribution in IBD vs Control

^b^Eadala reported breath tests in the bracketted space and genetic test results in the primary listed numbers, CC is the genotype for lactase non persitence from the C/T-13910 north European polymorphism

^**c**^Included information on previous surgery in Crohn’s disease. Mishkin reported no effect of surgery on LM. In these reports only Eadala reported a single patient after colectomy for ulcerative colitis

^d^Studies which included enough data on site of disease in CD and frequency of lactose maldigestion

^e^This study evaluated Jewish and non Jewish patients and controls

^f^Studies specifying ethnic make-up of patients and controls

^g^Kirschner et al. reported no effect of activity on LM

Eleven studies originated from countries with low risk for LNP [15, 23–32] and 4 specified ethnic make-up [15, 27, 30, 31]. Five reports were from moderate risk countries [33–37] and 1 was from a high risk country [38].

Twelve studies used a lactose challenge with measurement of BT [15, 24–27, 30, 33–38]. One of these also analyzed the C/T-13910 polymorphisms [24]. Two studies used LTT alone [31, 32]. Two evaluated jejunal biopsies [23, 28] and one used urinary lactose/L-arabinose sugar ratio to define LM [29]. Intestinal biopsies [39], BT and the LTT have been validated against genetic tests for the north European C/T- 13910 lactase polymorphisms [40].

#### Outcome of meta-analyses

**Fig. 2:**
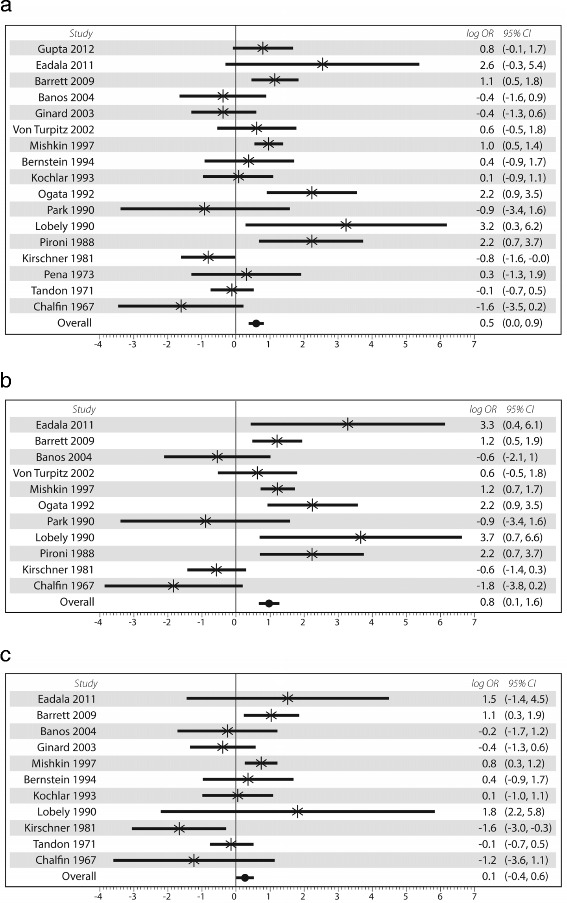
**a** Forest plot of 17 articles which evaluated indirect tests of lactose maldigestion in patients with inflammatory bowel diseases compared with controls. The 4 indirect tests were the hydrogen breath test, lactose tolerance test proximal small bowel biopsy and sugar urinary ratio test. **b** Forest plot of 11 studies evaluating only Crohn’s disease using all type of tests. **c** Forest plot of 11 studies evaluating only ulcerative colitis using all type of tests

The OR for LM in IBD vs. in controls including the 4 indirect tests was 1.61 (95 % CI: 1.00–2.57), *p* = 0.048. Heterogeneity was substantial at I^2^ = 69.3 %. In subgroup analyses, for CD specifically the outcome was still statistically significant with an OR of 2.29 (95 % CI:1.09–4.80, *p* = 0.03, I^2^ = 74.8 %), but did not reach statistical significance for UC (OR = 1.14, 95 % CI:0.69–1.86, *p* = 0.62, I^2^ = 53.8 %). Fig. 2 shows the forest plots with log OR using all type of tests.

**Fig. 3:**
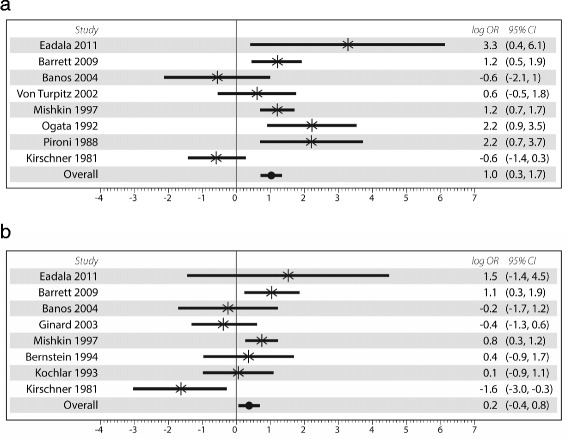
**a** Forest plot of 8 studies evaluating only Crohn’s disease using breath test. **b** Forest plot of 8 studies evaluating only Ulcerative colitis using breath test

Subgroup analysis using BT alone showed similarly that LM was only significant in CD (OR = 2.35, 95 % CI:1.21–4.57, *p* = 0.012, I^2^ = 74.1 %), but not in UC (OR = 1.21, 95 % CI:0.67 – 2.18, *p* = 0.53, I ^2^ = 59.8 %). Fig. 3 shows the forest plots with log OR using breath test only. LTT did not show any statistically significant differences in CD (*n* = 2 studies, OR = 1.0 (95 % CI:0.03–33.3)) or UC (*n* = 2 studies, OR = 0.84 (95 % CI:0.46–1.54)) [data not shown].

**Fig. 4:**
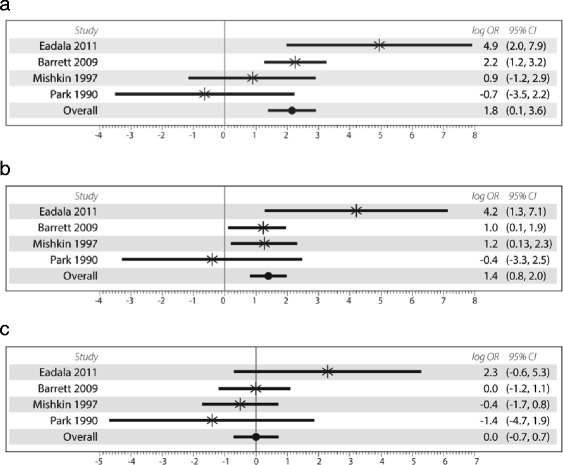
Forest plots of studies which divided Crohn’s disease patients by site of dominant disease using any test and including patients only from low risk for lactase non persistent status compared with healthy controls. Figure **a**, represents analysis of small bowel only, Figure **b** represents analysis of terminal ileum and colon, Figure **c** represents colon only

Sub-analysis of CD sites were analyzed in relation to SB, TiC or Colon. When all populations were included, site impact was not significant [SB: OR = 2.53(95 % CI: 0.45 – 14.3); TiC: OR = 1.42 (95 % CI:0.35 – 5.83); Colon: OR = 1.42 (95 % CI:0.82 – 2.46)], based on 5 studies [15, 24, 25, 28, 30]. However, when low risk populations were analyzed independently, SB and TiC sites were significantly associated with LM whereas Colon was not [SB: OR = 6.2 (95 % CI:1.01 – 35.1), *p* = 0.039, I^2^ = 65.3 %; TiC: OR = 4.2 (95 % CI:2.26 – 7.66), *p* < 0.0001, I ^2^ = 49.1 %; Colon: OR = 1.01 (95 % CI:0.49 – 2.06), *p* = 0.307, I^2^ = 16.8 %] [15, 24, 25, 28] (Fig. 4).

The effect of disease activity on LM status was conflicting, with some studies showing an effect [23, 26, 36], while another showed no effect in either UC nor CD [30]. Surgical history was not found to affect LM status in CD [15].

No publication bias was detected for the combination of all tests in IBD (*p* = 0.91), or individual papers on CD (*p* = 0.89) or UC (*p* = 0.37) using Egger’s test. No publication bias was detected for low-risk group CD tests (*p* = 0.51).

#### Description of studies not included in meta-analysis

There were a total of 9 studies of LM or LI that were excluded from the meta- analysis because no specific controls were provided. Outcomes were compared to nationally recognized frequency of LNP status or they described other aspects of tests [41–49]. Wiecke et al. examined jejunal biopsies from 65 children [mean age 14 range 3–18 years] with a number of gastrointestinal diseases and found low lactase levels in IBD, however these similar to national expectations [30–35 % LNP] [41]. In a large number of patients who underwent BT, Huppe et al. found the frequency of LM in 124 CD patients to be comparable with population data, but failed to explore effects of site, disease activity or resection extent on LI/LM. In 53 UC patients LM rates were significantly lower [3.8 % vs about 20 % of the German population] [42]. This finding was also reported by Mishkin et al. [15]. In a double-blind crossover study of 39 UC patients, BT outcomes were similar to Mexican population rates [46 % vs about 50 –70 %] [43]. In 2 studies from Denmark, (national LM prevalence, 5–6 %), performance of an LTT showed no difference from expected rates [6 % CD, 9 % UC] [44, 45]. However one reported higher LM rates with activity of IBD [44], while the other found no relationship other than ethnic distribution in UC patients [45]. Nevertheless a lactose free diet seemed to benefit patients without proof of LM [44].

Three other studies were identified. In one, concentrations of urinary lactose/raffinose were increased in 19 % of CD patients but no other specific details were given [46]. Two, studies reported outcomes of jejunal biopsies. Dunne et al. reported that small bowel concentrations of lactase and brush border surface were reduced in patients with CD, while levels in UC were comparable to controls [47], This was confirmed in another study of UC patients [48]. One study addressed symptoms only but these were not lactose related [49].

#### Milk and lactose intolerance and lactose sensitivity

Thirteen studies (excluding those dealing with nutrition because they describe different aspects) alluded to symptoms of milk or lactose intolerance [MI and LI respectively], [24, 26, 27, 30, 31, 33–37, 42, 44, 49]. In 7 of these, the terms LI and LM were interchangeable suggesting that symptoms during testing might reflect daily LI [27, 30, 31, 33, 34, 36, 42]. In 4 studies LI was used to define LM [27, 34, 36, 42]. LI and LM were more frequent in pancolitis than in left-sided colitis or proctitis, and more with disease activity [36]. LI occurred more frequently with small bowel CD or higher loads of lactose [37]. In 2 studies, MI was higher in CD [26] or UC [27] than in controls. However the rate of MI correlated with duration of disease rather than location, or resection length in CD [26].

Dissociation between LI and LM was noted in 5 studies [24, 26, 27, 33, 37]. In particular Eadala et al. noted a discrepancy between the frequency of genetic LNP in patients and controls compared with the prevalence of lactose sensitivity which reached 70 % in IBD [24]. They also noted a discrepancy between the results of genetic tests and BT, with positive BT tests occurring more frequently in patients. This observation was also noted by Barrett et al. [25], where ileal disease produced more frequent positive tests than ileocolic or colonic involvement. Pironi et al. recorded more frequent LM in operated CD compared with controls yet LI occurred in only 3 of 11 patients with operations [37].

#### Articles dealing with nutritional impact of dairy foods

A total of 14 articles were reviewed primarily for nutritional effects [50–63]. Descriptions of these publications are presented in Table 2. Two were epidemiological, showing a rise in incidence of IBD correlating with rising consumption of western type diet, particularly animal protein including DFs [50, 51]. In 2 cross sectional studies, consumption of 1.25 L of milk /week was beneficial in reducing symptoms in UC [52] while milk and yogurt reduced risk of CD in another study [53]. A prospective study suggested that pasteurized milk reduced risk for CD [54]. The most recent and largest prospective cohort study supported the observation that the highest quartile of milk intake significantly reduced CD risk. There was also an overall significant trend for reduced UC if data were analyzed 3 years after commencement of the study [55]. Four studies with different methodologies evaluating DFs among a general diet in IBD, did not find any statistically significant impact [56–59]. Four studies used questionnaires with different intended outcomes regarding role of DFs in IBD. One reported increased flares with DFs [60], while 3 reported reduced intake of milk and DFs [61–63]. In the report by Jowett restrictions were supported by professional advice [61] while symptomatic, active IBD patients were more likely to withhold DFs in another study [62].

**Table 2:** Studies reporting on the impact of dairy foods on inflammatory bowel diseases (IBD). DF = dairy food, Inc = incidence

Author	Type of Study	Crohn’s Disease (N)	Ulcerative Colitis (N)	IBD (N)	Healthy Control (N)	Outcome
Kitahora [50]	Epidemiology	-	10819	-	-	Positive correlate. with UC inc
Shoda [51]	Epidemiology	292	-	-	-	Inc high correlate with DF in univariate analysis
Magee [52]	Prospective Cross-section	-	81	-	-	1250 ml/week protective
Octoratou [53]	Prospective Cohort	28 new 30chronic	-	-	38	Milk and yogurt protective
Abubakar [54]	Case-control	218	-	-	812	Drinking pasteurized milk protects
Opstelten [55]	Cohort	110	244	-	401,326	DF protect CD 0.3 CI (0.13–0.65) UC diagnose >3 years Significant trend 0.04
Spehlmann [59]	Twin Study	-	-	512 Twins 1 with disease	207 and (392 non twin IBD)	DFs no effect
Jantchou [57]	Prospective Cohort	33	43	-	67504	DF no effect. Animal protein + correlate
Reif [58]	Case-control	33	54	-	144	Pre Disease no effect of lactose
Jowett^a^ [56]	Prospective Cohort	-	183 (52 % relapse)	-	-	No effect 1.17 CI 0.53–2.5 Medium intake no effect with high intake
Joachim [60]	Prospective Cross-section	33	27	-	-	DFs prominent in relapse
Jowett [61]	Prospective Cohort	-	183 (52 % relapse)	-	-	68 % diet thought important, most restricted DFs
Vernia [63]	Case–control	91	96	-	420 and (276 other diseases)	Females with CD and UC had significantly lower Ca intake than recommended
Brazil Lopes [62]	Cross Section	21	44	-	-	64.7 % restricted DFs

^a^Jowett et al. published 2 papers on the same population. One showing no impact of Dfs on UC and the second evaluated patients’ beliefs on effects of diet on UC

From papers reviewed for lactose maldigestion, Gupta et al. found that despite similar rates of LM, IBD patients restricted DFs [33]. Bernstein et al. noted that DF restriction by UC patients or physician advice was based solely on presence of disease [27]. Finally in a large study of CD patients, it was argued that a high fat content in DFs is likely the source of symptoms [49]. Self-reported LI and active disease were the most important patient reported reasons for DF restriction [24, 49, 62, 63].

Dietary advice included a milk free diet in UC, regardless of LM status [44]. Two suggested.

DF restriction with disease activity only [26, 36]. Five suggested to restrict if positive LM status is established [23, 28, 35, 41, 48], while 4 suggested that there was no need to restrict DFs in IBD at all [24, 27, 37, 49].

## Discussion

Relationships between IBD and DF suggest that rates of LM largely reflect ethnic backgrounds of patients. Activity of IBD and small bowel involvement in CD increases LM rates. Symptoms of LI during tests may not reflect daily DF reactions. Consequences of true or self perceived LI may impact on DF consumption which may have variable outcomes.

### Lactose maldigestion in IBD

In the mid 20th century, milk protein allergy was considered a possible cause of UC [64]. As well in the early 1960s reports emerged showing that intestinal lactase levels were diminished in UC and were accompanied by self-restricted and physician-advised reduction of DFs in IBD [65, 66]. The impact of ethnicity was not yet proven.

The meta-analysis on LM rates does show a statistically significant increase in prevalence. However, analysis of CD and UC independently reveals that the outcome is driven by CD with small bowel CD involvement. Sub analysis of BT also follows the overall pattern suggesting that LM is either secondary to mucosal disease, motility disorder or bacterial overgrowth not necessarily genetics [67]. In other cases ethnic distributions account for the frequency of LM. These results should be interpreted with some caution in light of moderate or high heterogeneity encountered.

The notion that IBD rates differ between LP and LNP populations rests on epidemiological correlations between IBD and population distributions of LP and LNP [8]. The outcome of the meta-analysis suggests that LP and LNP persons may be equally affected by IBD.

There are only few studies examining possible risks of different lactase alleles in IBD. Eadala et al. evaluated C/T-13910 polymorphism in a group of patients from Wales and found a 6 % rate of CC (LNP) genotype among 165 Welsh IBD patients [24]. This frequency is close to the national rate. Earlier reports were conflicting however. Buning et al. did not find any statistically significant differences in frequency of IBD among German patients with CC genotype [68]. Elguezabal et al. could not confirm increased TT/CT genotypes in Spanish patients [69]. However an earlier study from Spain [70] and one from New Zealand [71] did find increased prevalence of Crohn’s disease in TT genotype (LP phenotype) persons.

The current observations however can’t rule out different rates or delay in disease development between LP and LNP. For example IBD rates are different between Indigenous populations and Caucasians described both in Canada [72] and New Zealand [73]. In both areas Indigenous people are predominantly LNP and Caucasians are predominantly LP.

### Symptoms of LI

It is no longer accurate to equate LI with LM. The reason for this is that studies of LI in patients with irritable bowel syndrome (IBS) showed similar frequency of symptoms whether they were LNP or LP phenotype [74, 75]. Another reason is the ability of LNP persons to adapt to continued lactose consumption [76]. Lactose in LNP/LI persons induces symptoms (LI) through a metabolic effect on the microbiome [77]. In LNP persons, continued ingestion of sufficient lactose will lead to microbiome adaptation resulting in altered metabolomes as well as reduced test outcomes for BT [78].

However ability to adapt to lactose in IBD is unclear and there are no formal trials. Using lactulose, a disaccharide with similar properties to lactose failed to show adaptation compared with healthy controls [79]. Pironi et al. however may have detected microbial adaptation since despite increased LM status only 8 % of CD patients were also LI [37].

There are two other factors which may alter symptoms to lactose/DFs. As in the case of the study by Nolan –Clark, DFs containing fats may actually be the prime cause of symptoms [49], a possibility also stressed by Mishkin [12]. As well, a role of FODMAPs driving food sensitivities needs to be considered [80]. Restriction of these may reduce symptoms in IBD [81], since irritable bowel syndrome is frequent in IBD even in remission [82]. However, in this paradigm, genetics of lactase may not play a role. In studies reviewed, LI and LM were interchanged in several studies [27, 30, 31, 33, 34, 36, 42] and Eadala introduced the concept of lactose sensitivity [24]. They reported the highest symptom rate of any study, independent of genetic analysis and with discrepant outcomes with BTs [24]. The reasons for these observations aren’t clear and the study was criticized on methodological grounds [83]. This review in the end doesn’t allow a true estimate of the frequency of LI in IBD patients. Activity of disease, site in CD and surgical resections variably affected outcome. The overall impression is that self reported LI along with counseling led to DF consumption restrictions.

### Health related effects of lactose/dairy foods in IBD

Specific impact of milk and DFs consumption on IBD has not been studied as extensively as on other diseases (reviewed in [84]). In general there are 3 topics to consider: first is the relationship of DFs to risk of IBD, second DFs impact on IBD relapse rates, finally possible risks of dietary DFs restrictions.

While 2 epidemiological studies [50, 51] suggested a positive correlation of increasing DFs intake with increasing incidence of IBD, remaining studies suggested possible protection by DFs [52–55] or no effect on IBD [56–59]. Further studies are needed to verify protective outcomes. However there is a hint that an ecological fallacy type relationship between DFs and IBD exists. In this situation, observed ecological relationships between disease and target variables are opposite to those expected at patient level studies. A similar paradigm occurs between DFs and colorectal cancer [8, 85].

The second topic that DFs aggravate established IBD is not clear. One study reported that milk and DFs intake were associated with flares [60] and this is supported in a study of food groups in patients with UC [86]. However in a large review, of effects of general diets in IBD, no convincing evidence was found to show that any nutrient induced flares [7]. The presence of IBS and role of FODMAPs contributing to symptoms may cloud the issue [82].

The third topic is whether DF restriction has any negative impacts. In the general population, an NIH conference on LI concluded that the main health hazard is the improper withdrawal of DFs. Benefits from DFs were stressed [87]. Among these, bone health, better control of hypertension [88], weight gain [89, 90] and a reduced risk for colorectal cancer either through calcium or vitamin D is oberved [91].

In IBD, osteopenia and osteoporosis are consequences of chronic inflammation and medications [92–94]. The role of calcium and DF intake in IBD-related bone disease is unclear [95–97] or controversial [98] but intuitively is still important.

Colorectal cancer is increased in IBD colitis and may be linked with chronic inflammation also [99]. Calcium and vitamin D may be protective both for cancer [100, 101] and provide anti-inflammatory effects [102].

Risks of cardiovascular complications may be increased in IBD [103] and calcium may contribute by reducing arterial stiffness [88]. Evaluation of the specific impact of DFs in IBD require further evaluation.

There are limitations to this review. Conclusions from meta-analyses are as accurate as the papers reviewed. Although the quality of most studies was rated as adequate, the period spans 50 years with the majority of studies being older. The period of study includes 4 modalities of lactase assessment and conceptual changes in the genetics of lactase as well as concepts related to symptoms. These variations, as well as inclusion of different populations, and the few number of studies may well account for heterogeneity. However, in the studies reviewed for meta-analysis patients were always studied in parallel with healthy controls, and the outcome sought was an objective prevalence of LM in IBD regardless of method of ascertainment. To our knowledge, this is the only review to date which derives conclusions based on the available literature.

## Summary and conclusions

It is suggested that LM in IBD is determined by ethnicity in most cases of UC and CD. In CD small bowel involvement can produce secondary LM in LP. Although unproven, activity may also impact on LM. LM can aggravate LI but self reported LI or the overlap with FODMAP confound DFs specific role. Moreover there is emerging suggestive evidence that DFs may have benefits in IBD and restriction may impact unfavorably. Further work is needed to evaluate the role of DFs in IBD as well on methods to avoid their restriction.
